# Signal-On Fluorescence Biosensor for Detection of miRNA-21 Based on ROX labeled Specific Stem-Loop Probe

**DOI:** 10.5812/ijpr-144368

**Published:** 2024-03-30

**Authors:** Somayeh Heidarian, Laya Takbiri Osgoei, Shohreh Zare Karizi, Jafar Amani, Sedigheh Arbabian

**Affiliations:** 1 Department of Biology, Faculty of Biological Science, North Tehran Branch, Islamic Azad University, Tehran, Iran; 2 Department of Microbiology, Faculty of Biological Science, North Tehran Branch. Islamic Azad University, Tehran, Iran; 3 Department of Biology, Varamin Pishva, Branch, Islamic Azad University Pishva, Varamin, Iran; 4 Microbiology Research Center, Systems Biology and Poisonings Institute, Baqiyatallah University of Medical Sciences, Tehran, Iran

**Keywords:** Nano Biosensor, MicroRNA, Colorectal Cancer, Carbon Nanotubes

## Abstract

**Background:**

The abnormal expression of microRNA (miRNA) influences RNA transcription and protein translation, leading to tumor progression and metastasis. Today, reliably identifying aberrant miRNA expression remains challenging, especially when employing quick, simple, and portable detection methods.

**Objectives:**

This study aimed to diagnose and detect the miR-21 biomarker with high sensitivity and specificity.

**Methods:**

Our detection approach involves immobilizing ROX dye-labeled single-stranded DNA probes (ROX-labeled ssDNA) onto MWCNTs to detect target miRNA-21. Initially, adsorbing ROX-labeled ssDNA onto MWCNTs causes fluorescence quenching of ROX. Subsequently, introducing its complementary DNA (cDNA) forms double-stranded DNA (dsDNA), which results in the desorption and release from MWCNTs, thus restoring ROX fluorescence.

**Results:**

The study examined changes in fluorescence intensities before and after hybridization with miRNA-21. The fluorescence emission intensities responded linearly to increases in miR-21 concentration from 10^-9^ to 3.2 × 10^-6 ^M. The developed fluorescence sensor exhibited a detection limit of 1.12 × 10^-9^ M.

**Conclusions:**

This work demonstrates that using a nano-biosensor based on carbon nanotubes offers a highly sensitive method for the early detection of colorectal cancer (CRC), supplementing existing techniques.

## 1. Background

Colorectal cancer (CRC) is identified when unusual growths develop in the inner and outer layers of the colon and rectum. These growths, known as polyps, can be categorized as either adenomatous or hyperplastic. While adenomatous polyps are benign, cancer cells can spread to the surrounding areas, infiltrating the bloodstream and lymphatic vessels (1). The International Agency for Cancer Research (IARC) identifies this diverse type of cancer as the third most common worldwide. Additionally, according to 2018 World Health Organization (WHO) data, it ranks as the second leading cause of cancer-related deaths and is the most lethal form of cancer (2). Furthermore, between 70 and 80 percent of cases are sporadic, with the remaining around 10 percent being hereditary CRC (3). The five-year survival rate for patients with distant metastases is extremely low at 10% (4). Early detection of CRC can increase the five-year survival rates from approximately 13% in cases of advanced-stage metastatic cancer to 90% in early-stage diseases (5, 6). Therefore, early identification of CRC can reduce mortality related to the disease and pave the way for more treatment options and strategies. Over the past decade, several CRC screening methods have been developed, including sigmoidoscopy, CT colonography (CTC), colonoscopy, the fecal occult blood test (FOBT), stool DNA test, double-contrast barium enema, and colonoscopy (7). FOBT, which tests for hemoglobin in feces using an antibody, is the most commonly used and cost-effective method but suffers from a high rate of false positives and negatives and limited sensitivity. In contrast, CTC, sigmoidoscopy, and colonoscopy offer more accurate direct visualization of lesions but require thorough bowel preparation, are more costly, and have lower participation rates (8, 9). Increasing research suggests that tumor markers, which can be proteins, enzymes, genes, gene products, specific cells, or hormones, might be detected in bodily fluids or tissues, indicating the presence of cancer. On the other hand, methods such as (enzyme-linked immunosorbent assay (ELISA) (10), immunohistochemistry (11), IHC (11), radioimmunoassay (12), fluorescence (13), chemiluminescence (14), electrophoresis (15), and polymerase chain reaction (PCR) (16) have been developed for CRC detection. While these approaches can yield reliable results, they come with several disadvantages, such as lengthy processes, complex operation procedures, and a high demand for sample volume. Additionally, the trace amounts of biomarkers present during the early stages of CRC may not be detectable. Consequently, there is a need for a rapid, accurate, simple, and cost-effective method for biomarker identification to aid in the early detection and treatment of CRC.

In recent years, research has increasingly focused on circulating microRNAs (miRNA), which have been suggested as valuable diagnostic biomarkers for various types of cancer (17, 18). MicroRNAs, a significant subset of small non-coding RNAs (ncRNAs), belong to a family of short (19 - 25 nucleotides), single-stranded, non-coding RNAs that regulate protein synthesis by binding to the 3' UTR of target mRNAs (19). MiRNAs are considered effective biomarkers for CRC detection because they can be found in bodily fluids and exhibit high stability in the presence of RNase activity, boiling temperatures, extreme pH levels, multiple freeze/thaw cycles, and long-term storage (20). The role of miRNA as epigenetic factors in the pathogenesis of CRC has been evaluated and confirmed in numerous studies, highlighting their potential as biomarkers for the diagnosis of surgically curable stage II CRC. The main challenge with stage II CRC is the risk of disease recurrence and increased mortality. miR-21 stands out as a crucial biomarker for stage II CRC and is among the microRNAs (miRNAs) extensively researched across various cancers. Studies have shown that miR-21 is significantly overexpressed in a broad spectrum of cancers, including esophageal, gastric, breast, colorectal, hepatocellular, pancreatic, as well as in glioblastoma, leukemia, B-cell lymphoma, cholangiocarcinoma, lung cancer, and squamous cell carcinomas of the cervix, tongue, neck, and prostate. This widespread elevation suggests miR-21's key role in the onset, development, and spread of many types of cancer. As an oncogenic microRNA, often referred to as "oncomiR," miR-21 plays a critical role in regulating the cell cycle, apoptosis, migration, differentiation, and stem cell renewal. Many of its targets are involved in the initiation, transformation, invasion, and metastasis of cancers (21, 22). 

Nanomedicine has played a significant role in enhancing CRC detection, offering greater sensitivity, cost-effectiveness, and a reduction in the over-diagnosis and under-diagnosis of cancer (23). Meanwhile, nanomedicine has surpassed endoscopic examinations for the morphological inspection of the intestinal epithelium, which cannot identify colon tumors at the molecular level (24). Nanotechnology focuses on manipulating materials at the nanoscale to detect malignant or precancerous cells at an early stage. Recent advancements in nanotechnology have facilitated the creation and application of a diverse array of nanostructures with exceptional chemical, physical, and mechanical properties for use in biosensing devices (25).

Biosensors are powerful tools for the rapid, precise, and sensitive detection of biological substances across a broad spectrum of applications (26). The principle of biosensor identification is based on the interaction between analytical and biological systems (27). Carbon nanostructures, specifically carbon nanotubes (CNTs), have become essential elements in biosensor designs due to their outstanding electrical, optical, and mechanical properties (28). Defined as seamless cylindrical structures that can consist of one or several layers, either with open or closed ends, carbon nanostructures (CNTs) are categorized into single-walled carbon nanotubes (SWCNTs) and multi-walled carbon nanotubes (MWCNTs). The significant specific surface area of CNTs facilitates the immobilization of various functional components, including receptor molecules, simplifying biosensing applications. Additionally, CNTs possess unique intrinsic optical properties like strong resonant Raman scattering and photoluminescence in the near-infrared (NIR) region, making them exceptionally suited for biological detection (29). A wide array of CNT-based biosensors has been developed to detect various cancer biomarkers by attaching DNA, aptamers, antibodies, peptides, proteins, or enzymes (30).

Numerous studies in recent decades have underscored the role of microRNAs in carcinogenesis and tumor progression (17, 19, 31). Among all types of non-coding RNAs (ncRNAs), microRNAs have garnered significant attention due to their frequent dysregulation in CRC (18). Incorporating cancer-associated miRNAs, specifically miRNA-21-5p, has significantly improved the diagnostic accuracy of the APC gene mutation panel in circulating cell-free tumor DNA (ctDNA) for CRC detection (32). 

## 2. Objectives

A nano-biosensor for identifying miRNA-21-5p, aimed at diagnosing CRC, was developed using MWCNTs and DNA tagged with ROX.

## 3. Methods

### 3.1. MiRNA Isolation Method from Clinical Samples

After obtaining informed consent, 2 ml of blood was drawn from each patient and collected in tubes containing EDTA. These samples were taken from five male patients aged between 65 and 71 years, all diagnosed with stage IV CRC. The cell-free plasma was then separated from the blood by first centrifuging at 2 000 × g for 10 minutes, followed by microcentrifugation at 1 1000 × g for 3 minutes. Following the manufacturer's guidelines, 200 µl of plasma from the cancer patients was utilized to extract microRNA using the miRNeasy Serum/Plasma Kit by Qiagen. The quantity of RNA was determined by measuring the absorbance at a wavelength of 260 nm using a spectrophotometer (UV-1800, SHIMADZU). The purity of the RNA was assessed using A260/A280 ratios.

### 3.2. Reagents

The specific probe for the miR sequence, '5-TAGCTCGGTCAACATCAGTCTGATAAGCTAAAC-3', along with its complementary target sequence, '5-TAGCTTATCAGACTGATGTTGA-3', and a three-base mismatched sequence, '5-TTGCTTGTCAGACTGATCTTGA-3' (mismatched bases underlined), as well as a non-complementary sequence, '5-GTAAGGCATCTGACCGAAGGCA-3', were synthesized by Bioneer, South Korea. The oligonucleotide sequences were purified using HPLC. All oligonucleotides were dissolved in deionized water to make 100 µM stock solutions and stored at -20°C.

The miR-21 probes were designed to target specific sequences at the 3' end of miR-21 in humans using Oligo Analysis Software version 7.60. The probe's 5' end was labeled with the ROX dye. The nanomaterials, including multiwall carbon nanotubes (MWCNTs) with a carbon purity greater than 95% (Cat#755125), were purchased from Sigma-Aldrich, based in the US. The carboxylated MWCNTs were initially subjected to a mixture of concentrated acids (HNO_3_:H_2_SO_4_ = 1:3) under ultrasonic agitation for three hours, followed by three washes with water.

### 3.3. Optimization of the Absorption of the ROX-Labeled miR-21 Specific Probe on the Surface of MWCNTs

Previous research has determined the optimal concentration for quenching fluorophore probes and creating fluorescent biosensors to be 1 mg/mL. The procedure involved combining 10 µL of the miR-21 probe (10 pM) with 15 µL of MWCNTs (1 mg/mL) in a final solution volume of 2 mL Tris-HCl (pH 7.4, 0.02 mM). The Tris-HCl solution was composed of Tris (hydroxymethyl) aminomethane, NaCl, KCl, and MgCl_2_, all dissolved in 100 ml of deionized (DI) water. The fluorescence emission was monitored at various time intervals.

To identify the ideal concentration for effectively quenching the fluorescence of the probe, different concentrations of MWCNTs were mixed with 10 µL of the miR-21 probe (10 pM). The optimal time for absorption was determined through fluorescence spectrometry to assess fluorescence quenching. The formation of MWCNT-probe conjugates was verified using energy-dispersive spectroscopy (EDX) and scanning electron microscopy (SEM) with a Zeiss-DSM 960A microscope. Fluorescence spectra measurements were conducted using a varian cary eclipse fluorescence spectrophotometer.

### 3.4. Detection of the miR-21-Specific Sequence by a miR-21 Probe-MWCNTs-Based Nanosensor

The evaluation of the hybridization response involved adding the matching target DNA to the ROX-labeled probe-MWCNT conjugate after its synthesis. To accelerate the reaction time during the initial phase, the fluorescence emission intensity was chronologically monitored. Subsequently, various concentrations of complementary DNA were mixed with the MWCNT-ssDNA conjugates to find the optimal concentration for the hybridization process, and the fluorescence intensity was measured at the ideal hybridization time. The sensitivity of the nanosensor was determined by monitoring the probe fluorescence emission across 10-fold serial dilutions (from 50 pg to 3.2 M) of synthetic complementary DNA of the miR-21 probe. The negative control comprised genomic DNA from healthy control samples.

### 3.5. Determination of LOD and LOQ

The limit of blank (LoB), limit of detection (LoD), and limit of quantification (LoQ) were established following specific protocols. The LoB was defined as the highest apparent analyte concentration observed in replicates of a blank sample containing no analyte, calculated as the mean blank plus 1.645 times the standard deviation of the blank (LoB = mean blank + 1.645(SD blank)). The LoD corresponded to the lowest concentration of the analyte that could be detected, determined as LoB plus 1.645 times the standard deviation of a low-concentration sample (LoD = LoB + 1.645 (SD low concentration sample)). Replicates of a sample known to contain a low concentration of the analyte were tested for this purpose. Finally, the LoQ was identified as the lowest concentration at which the analyte could not only be reliably detected but also meet some predefined criteria for bias and precision (33).

### 3.6. Detection Process Using Real-Time PCR

Stem-loop real-time RT-PCR was utilized to evaluate miRNA expression. The design for miR-21 stem-loop reverse transcription (RT) primers and amplification primers followed the method outlined by Huang et al. (23). Specific stem-loop RT primers enabled the generation of cDNAs from total RNA, with the miR-21 sequence being: '5′-GTCGTATCCAGTGCAGGGTCCGAGGTATTCGCACTGGATAC GACTCAACA -3′. The reverse transcriptase reactions included 10 ng of RNA sample, 60 nM stem-loop RT primer, 1 × RT buffer, 0.25 mM of each dNTP, 4 U/μL M-MLV reverse transcriptase (Promega, Madison, WI, USA), and 0.4 U/μl RNase inhibitor (Takara). The reactions (10 μL) were incubated in a GenAmp PCR System 9700 (Applied Biosystems, Foster City, CA, USA) at 16°C for 30 minutes, 42°C for 30 minutes, and 85°C for 5 minutes, with a final holding step at 4°C. Real-time PCR was conducted using a Thermal Cycler Dice real-time system TP800 (Takara). The universal reverse primer for miR-21 was '5′-CAGTGCAGGGTCCGAGGT-3′, and the specific forward primers were '5′-GCCCGCTAGCTTATCAGACTGATG-3′ (miR-21). A 25 μL PCR reaction mixture containing 1× SYBR premix Ex Taq mix (Takara), 2 μL RT products, and 10 nM of each forward and reverse primer was incubated in a 96-well plate at 95°C for 30 seconds, followed by 45 cycles at 95°C for 15 seconds and 60°C for 21 seconds. A dissociation step from 65 to 95°C confirmed the specificity of the amplification products. Threshold cycle data were derived using the second derivative max settings. The U6 gene served as an internal control for normalizing the levels of the target miRNA. The stem-loop reverse transcription (RT) primers and amplification primers for U6 were sourced from Ribobio Co., Ltd., in Guangzhou, China.

### 3.7. Statistical Analysis

All fluorescence measurement experiments were conducted in triplicate on one day and across three different days. The results presented in each figure are expressed as mean values ± standard deviation. All statistical analyses were carried out using the GraphPad Prism 9 statistical program. The statistical differences among the mean fluorescence emission values of the biosensor in reaction with the control and miR-21 were determined by one-way ANOVA, with statistical significance established at P < 0.05 and P < 0.01.

## 4. Results

### 4.1. Design Strategy

**Figure 1.**
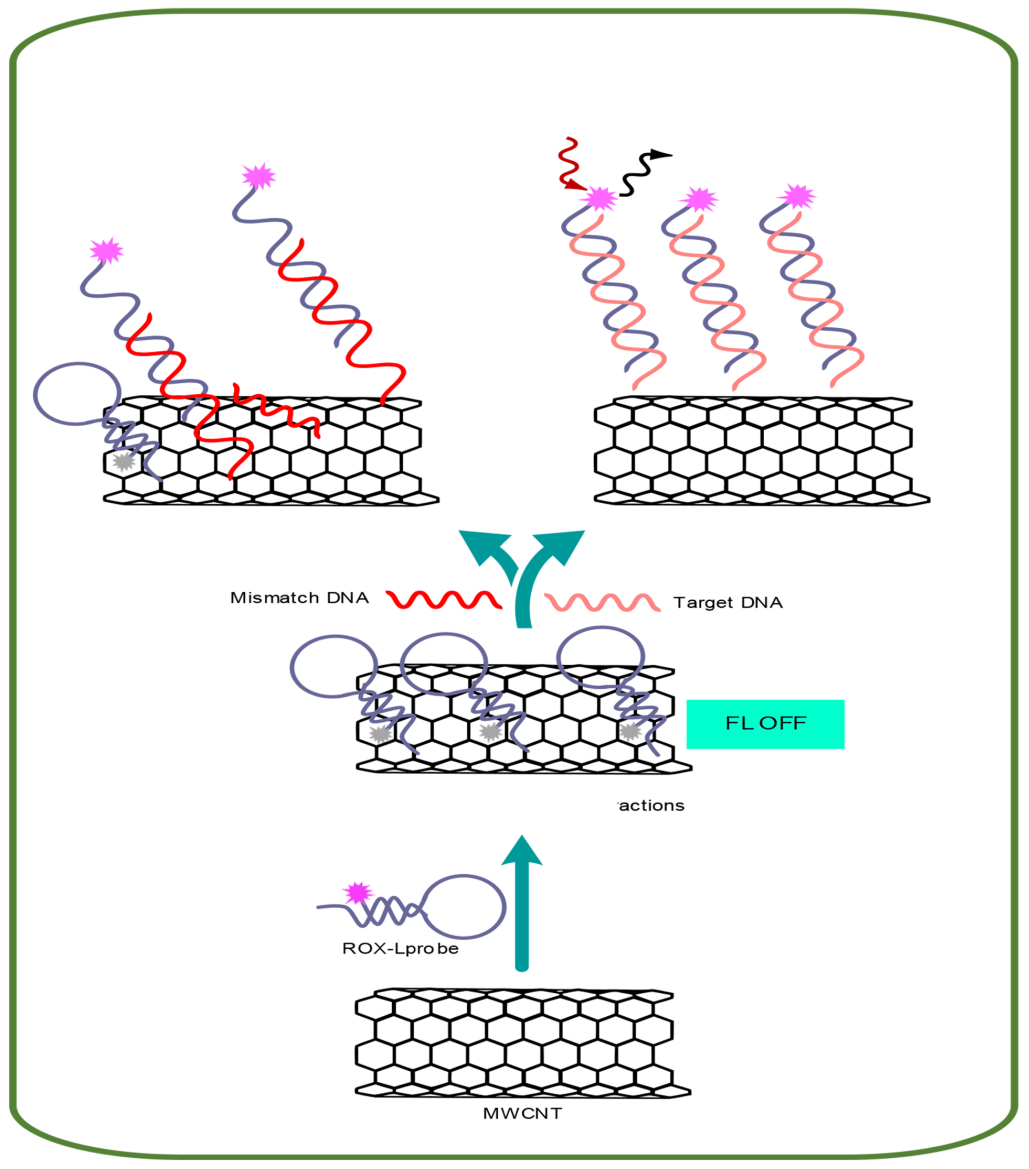
Schematic illustration of the multi-walled carbon nanotube (MWCNT)-based DNA fluorescent biosensor

The fundamental concept behind the proposed fluorescent nucleic acid sensing platform is depicted in Figure 1. This concept is based on the quenching of fluorescence due to the adsorption of a fluorescently labeled single-stranded DNA (ssDNA) probe (ROX-labeled probe) onto MWCNTs. Conversely, when complementary DNA (cDNA) is introduced, a double-stranded DNA (dsDNA) forms between a ROX-labeled probe and cDNA. This duplex is released from the surface of MWCNTs, resulting in the restoration of fluorescence emission.

**Figure 2.**
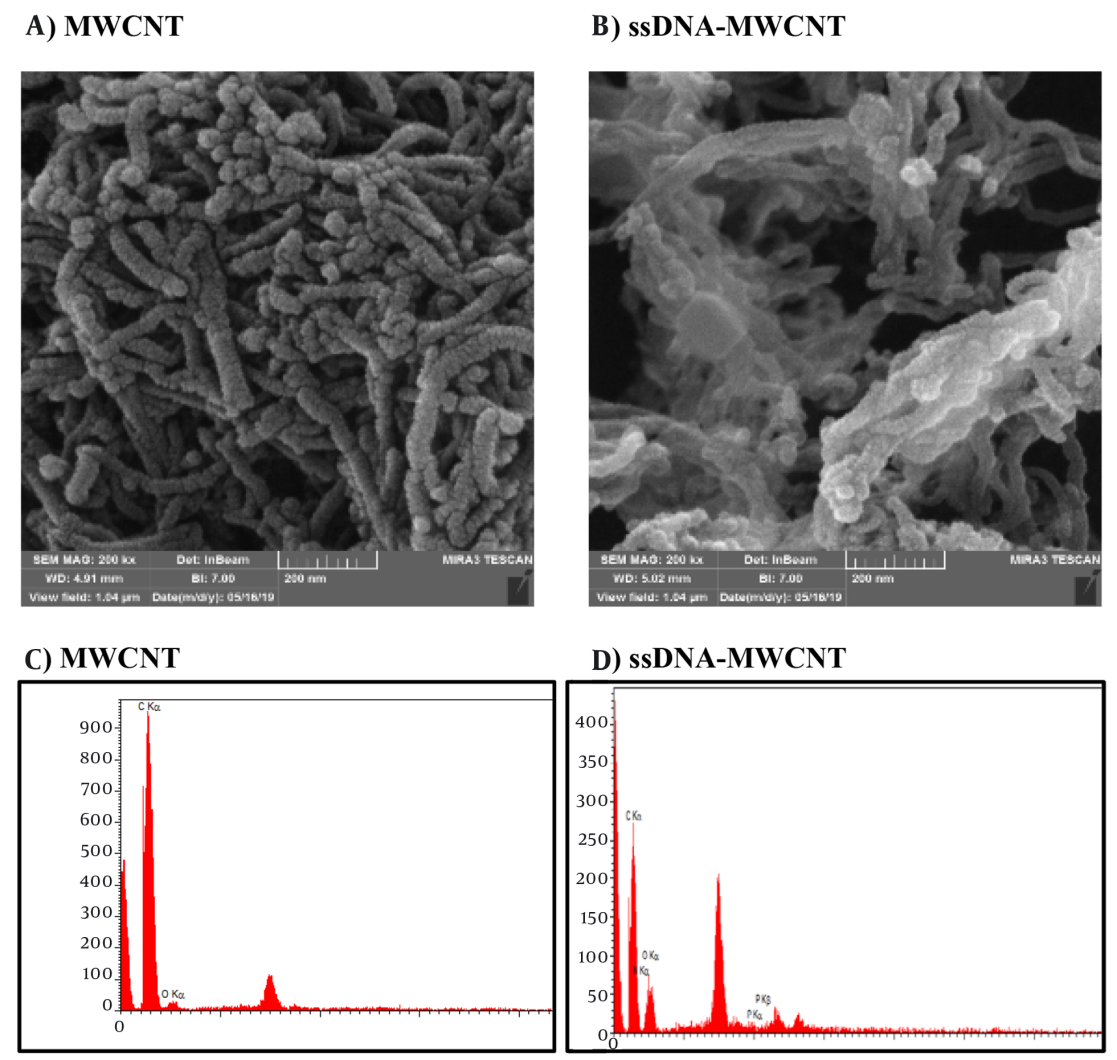
Scanning electron microscopy (SEM) image of multi-walled carbon nanotube (MWCNT) and MWCNT-ssDNA. A, SEM image of MWCNT; B, SEM image of MWCNT-ssDNA complex; C: Energy-dispersive spectroscopy (EDX) spectrum of MWCNT; D: EDX spectrum of material containing N and P elements in ROX labeled ssDNA-MWCNT conjugate.

SEM and EDX tests were employed to validate the creation of the MWCNT-ssDNA conjugates. Changes in the diameter and morphology of MWCNTs were observed in the SEM images of the MWCNTs/ssDNA probe conjugates (Figure 2A and B), indicating that the ssDNA probe was successfully adsorbed and stabilized by the MWCNTs. Energy-dispersive spectroscopy was utilized for elemental analysis. According to the results, the EDX spectrum displays strong peaks corresponding to carbon and oxygen elements before the attachment of miRNA to MWCNTs (Figure 2C). After the attachment of miRNA to the surface of MWCNTs, distinct peaks indicative of nitrogen and phosphorus elements in the miRNA strands appear in the EDX spectrum (Figure 2D). 

### 4.2. Analytical Characterization of the Designed Nanobiosensor in the Presence of miR-21

The hybridization reaction was conducted following the preparation and characterization of the MWCNT-ssDNA conjugate. Initially, the ROX-labeled probe, which emits fluorescence at 605 nm, experienced quenching upon its immobilization on the MWCNT surface. However, the fluorescence emission was restored due to complementary base pairing after the addition of the complementary sequence to the probe and the execution of the hybridization process. The analysis focused on the fluorescence emission spectra of an ssDNA probe tagged with ROX at the 5'-end, specific to the miR-21 sequence. In the absence of MWCNTs, the probe exhibited strong light emission at a wavelength of 605 nm. Nevertheless, the addition of MWCNT (40 µg) led to a significant reduction in fluorescence emission, with up to a 96% decrease in intensity. This significant quenching effect is attributed to the adsorption of the DNA probe onto the MWCNT surface through π → π* stacking interactions and hydrogen bonding (34).

**Figure 3.**
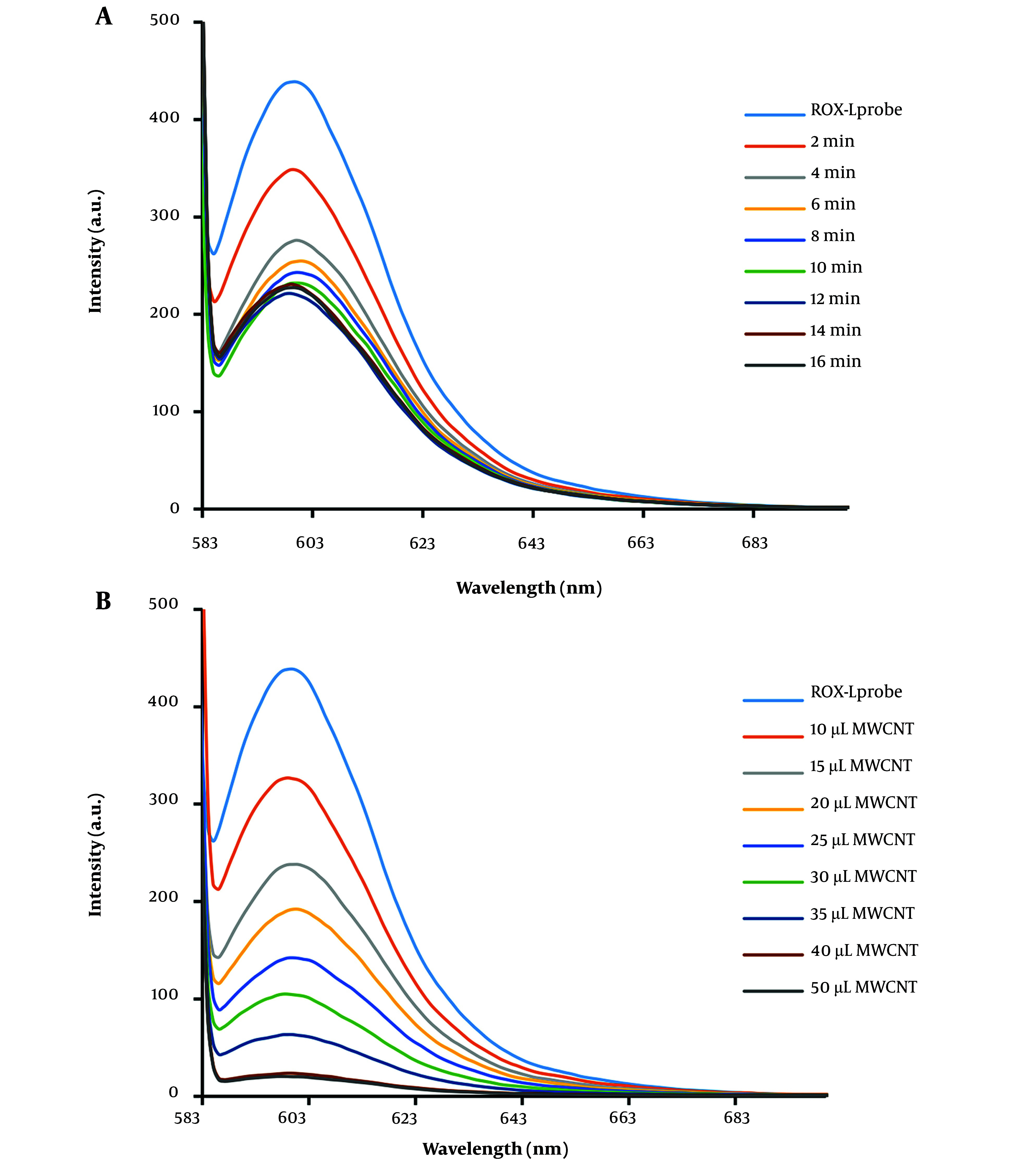
A, fluorescence emission spectrum of ROX-ssDNA in the presence of MWCNT at different time points. The intensity of fluorescence scattering decreased with increasing time, and after 6 minutes, no significant difference in fluorescence scattering intensity was observed. B, fluorescence spectrum of ROX-ssDNA in the presence of different concentrations (1 mg/mL) of MWCNT for complete extinction of fluorescence emission. As the amount of MWCNT increased, the intensity of fluorescence emission decreased, and after adding 40 µL of MWCNT nanoparticles, complete extinction of fluorescence emission was observed.

**Figure 4.**
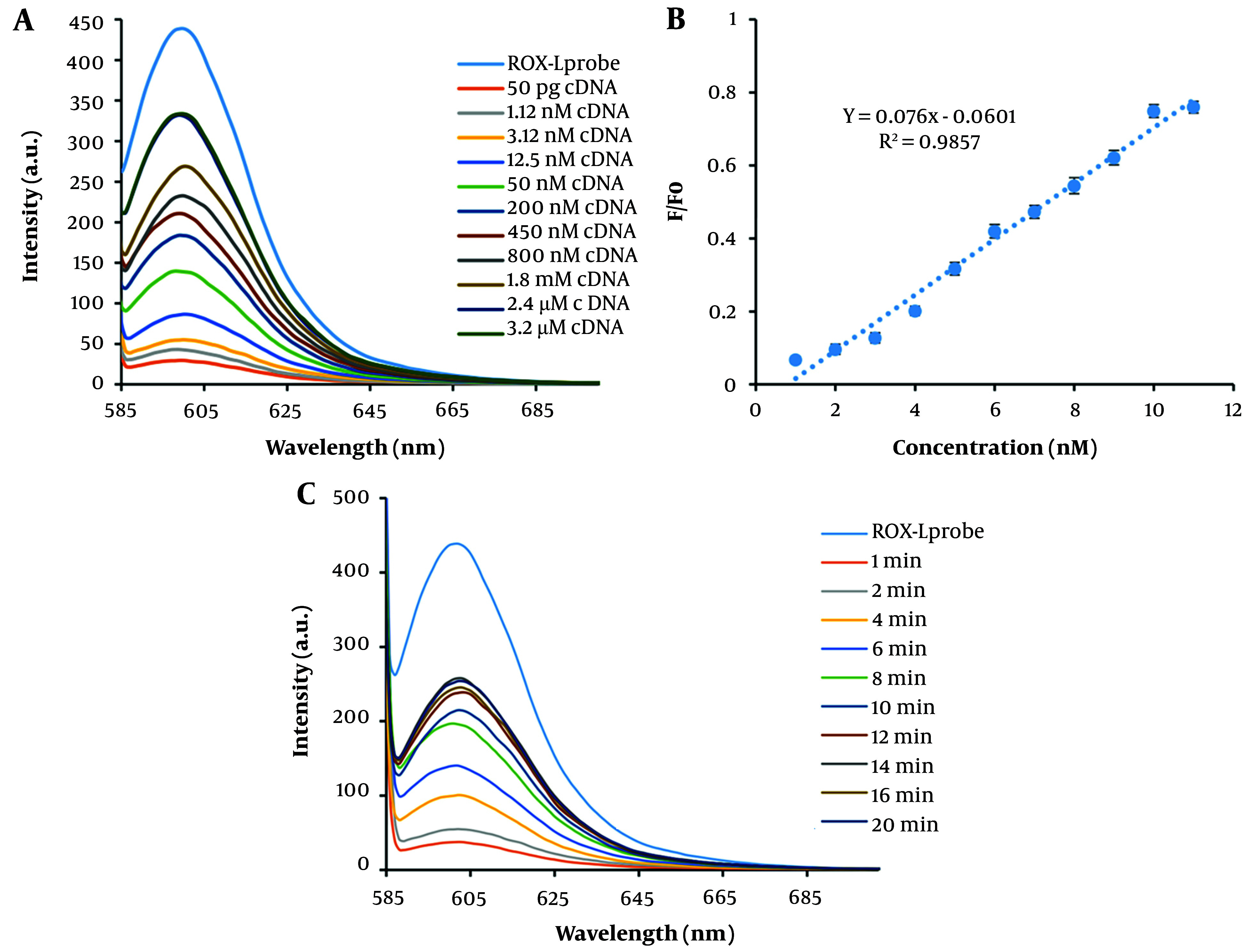
Fluorescence Emission Spectrum and Calibration Curve of Hybridization. A, fluorescence emission spectrum and a calibration curve of hybridization at different concentrations (50 pg, 1.12 nM, 3.12 nM, 12.5 nM, 50 nM, 200 nM, 400 nM, 800 nM, 1.8 mM, 2.4 mM, and 3.2 mM). B, target calibration curve. Corresponding fluorescence emission spectra in the presence of different concentrations of DNA target and calculation of the calibration curve. A linear correlation of 0.098 was obtained. C, fluorescence emission spectrum of the hybridization reaction at different times. The fluorescence emission intensity gradually increases with time and reaches its maximum value after 12 minutes. Therefore, 12 minutes was chosen as the optimal time for the hybridization reaction.

To optimize the absorption time of the DNA probe on MWCNT, the fluorescence emission was measured at various intervals in the presence of MWCNT. As a result, the fluorescence emission intensity decreased gradually, and an optimized absorption time of 6 minutes for the DNA probe on MWCNT was established (Figure 3A). Additionally, the impact of MWCNT concentration on the fluorescence emission intensity of the DNA probe was investigated. The results, depicted in Figure 3B, indicate that 40 µg of MWCNT is the optimal concentration for nearly complete quenching of the DNA probe's fluorescent emission (10 pM). Following the addition of cDNA at a concentration of 10 pM, the fluorescence spectrum of the MWCNT-DNA probe mixture was analyzed. Figure 4C illustrates that introducing cDNA led to an increase in fluorescence emission intensity at 605 nm. This increase can be attributed to the hybridization of the DNA probe with cDNA, resulting in duplex formation and subsequent detachment from the MWCNT surface. Measurements of various fluorescence emission spectra indicated that 12 minutes is the optimal duration for duplex formation (Figure 4C). Figure 4A and B display the fluorescence emission spectrum in the presence of different DNA target concentrations, demonstrating how fluorescence emission intensity escalates with rising DNA target concentration. According to the analysis, the relationship between fluorescence intensity and cDNA concentration was nonlinear, described by the equation y = -1.2031x + 11.986 with an R^2^ value of 0.7594. By examining the fluorescence response of the biosensor to cDNA and a mismatched DNA, a qualitative analysis was conducted to assess the selectivity of the miRNA sensing platform. The DNA biosensor's LOD and LOQ were determined to be 1.12 nM and 3.2 M, respectively, as shown in Table 1. 

**Table 1.** Comparison of Limit of Detection of miR-21 Biosensor and Other Nanobiosensors

Fluorescent Materials	Targets	Linear Interval, pM	Limit of Detection, pM	Reference
**Protonated phenyl-doped carbon nitride, ROX**	miRNA-224	10^3^ - 2 × 10^4^	200	(35)
**FAM, TAMRA**	miRNA-21	10^2^ - 2 × 10^4^	73	(36)
**NMM, DAPI**	miRNA-21	10 - 4.5 × 10^4^	3.1	(37)
**CDs, FAM**	miRNA-21	50 - 10^4^	1	(38)
**CdTe QDs, FCMMs**	let-7a	2 - 2 × 10^2^	0.1	(39)
**Boron-doped g-C3N4 nanosheets, Cu NCs**	miR-582-3p	0.2 - 1	0.049	(40)
**FAM**	miRNA-21	0.1 - 1 × 10^3^	0.1	(41)
**Hairpin structure molecular beacons**	let-7a	1 - 10^4^	0.0325	(42)
**MWCNTs@Au NCs, Atto-425**	miR-92a-3p	0.1 - 10	0.031	(43)
**In this study**	miRNA-21	-	1.12	-

**Figure 5.**
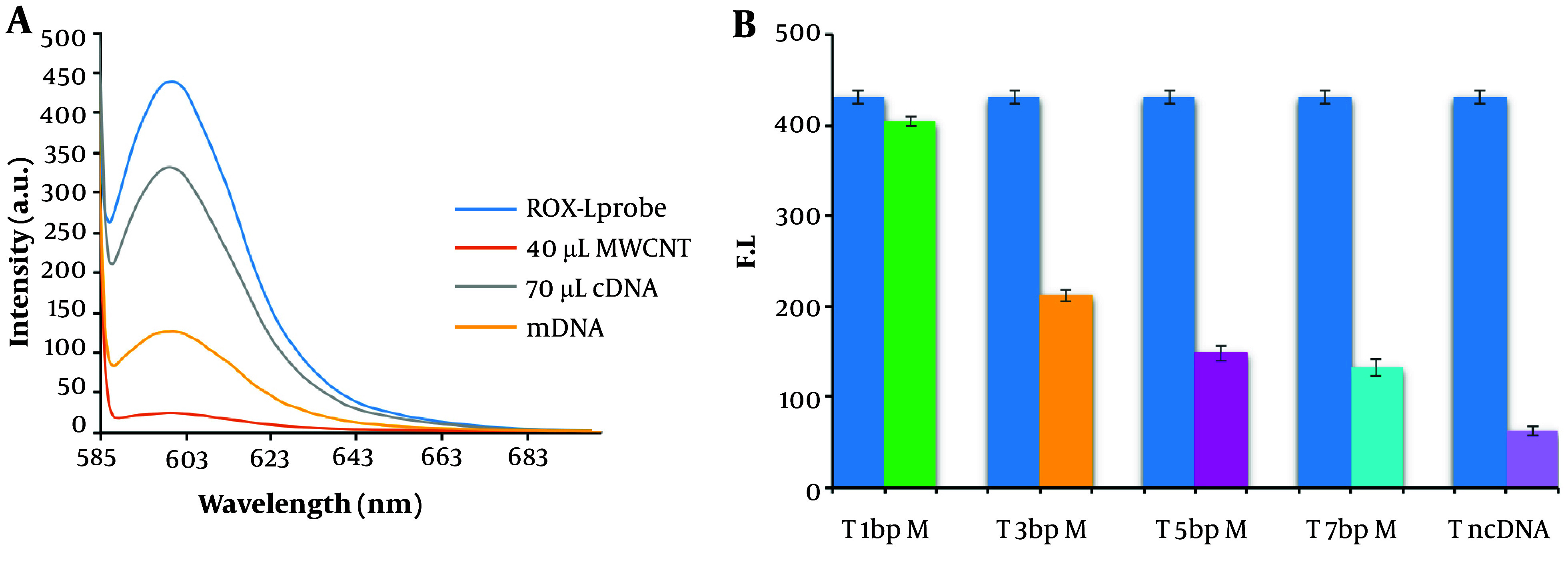
Analysis of biosensor selectivity. A, fluorescence spectra for evaluating the selectivity of the MWCNT-based biosensor. B, bar graph depicting the detection of cDNA (T: Target molecules) and mismatched DNA (mDNA) in the presence of single-, three-, and five-mismatch DNA targets.

**Figure 6.**
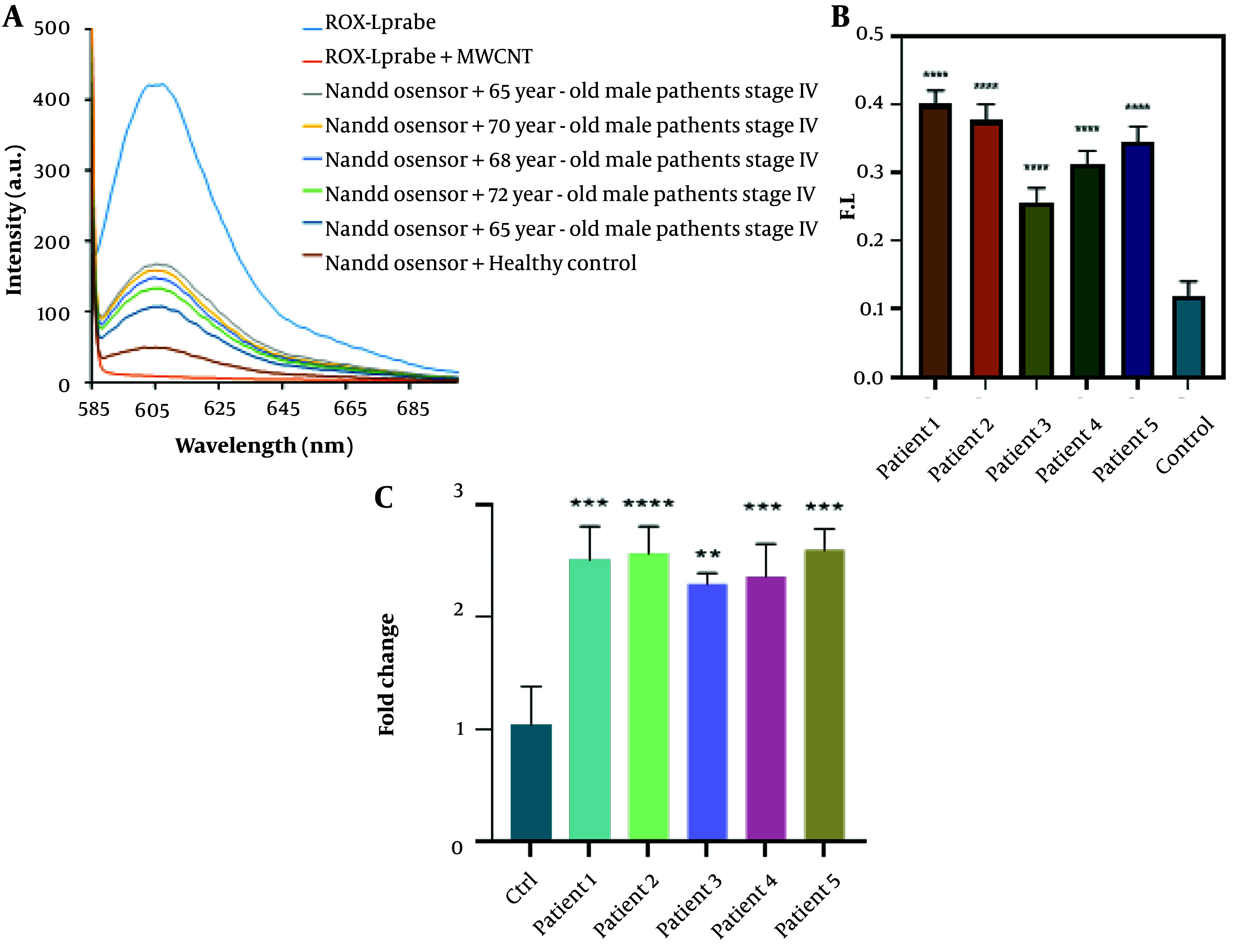
The fluorescence spectra and real-time PCR data were used to evaluate the applicability of the biosensor for detecting miR-21 in five different patient serum samples. Statistical analysis of the main samples is also presented. A, fluorescence spectra of the biosensor for miR-21 detection. Significant fluorescence restoration was observed in miRNA extracted from plasma samples compared to the control (normal sample) (P < 0.05). B, statistical analysis of the main samples. Non-parametric one-way ANOVA was performed for statistical analysis. Error bars represent the mean ± SD. *P < 0.05, **P < 0.01, ***P < 0.001, and ****P < 0.0001. C, bar graphs illustrating the expression levels of miR-21 in cancer patients and controls. miR-21 was significantly up-regulated (P < 0.05) compared to normal cells.

Figure 5 demonstrates that the biosensor's fluorescence signals in response to three-base mismatched DNA were 38.05% of those observed with the complementary DNA (cDNA). These results suggest that the nano biosensor specifically responded to the target cDNA, unlike other sequences. During the hybridization process for miRNAs isolated from the blood of cancer patients, an average fluorescence emission intensity of 142.6 was recorded, significantly higher than that of non-cancer individuals, which had an average emission intensity of 49.3 (Figure 6). The findings indicate that the presence of the probe-target sequence in blood samples from CRC patients (miRNA concentration extracted from plasma samples, 0.424 µg) resulted in significantly higher fluorescence emission in the hybridization reaction of miR-21.

### 4.3. Comparing the Novel Nanobiosensor with the Standard Real-Time PCR Method

The expression levels of all analyzed miRNAs were found to be significantly different between tumor and normal cells. Specifically, expression levels of miR-21 were upregulated in CRC cells by 1.5 times compared to normal cells (P < 0.05) (Figure 6C). The results obtained from real-time PCR are in agreement with those from the biosensor, mutually confirming the validity of each method.

## 5. Discussion 

Biosensor technology has seen significant advancements in the detection and diagnosis of biomarkers for CRC over recent decades (44). Biosensors are generally classified into three main types: Electrochemical, mechanical, and fluorescent (45). Fluorescence biosensors are non-invasive analytical tools designed to detect biomolecules in biological samples by sensing the absorption of electromagnetic radiation by fluorophores or fluorescently labeled molecules (46). These biosensors have been developed using a range of nanoparticles, including carbon, gold, and silver nanoparticles. Fluorescent sensors are highly valued in clinical diagnostics due to their exceptional selectivity, sensitivity, and rapid response times (47). The proposed platform for nucleic acid detection utilizes the principle of fluorescence quenching, which occurs when a fluorescently labeled ssDNA probe, known as the ROX-L probe, binds to MWCNTs, as illustrated in the Graphical Abstract. The fluorescence of the ROX-L probe is preserved after the prior hybridization with its complementary DNA target to form dsDNA, owing to the weak interactions between dsDNA and MWCNTs that keep the dsDNA away from the MWCNT surface. The interactions between DNA and MWCNTs are predominantly governed by electrostatic repulsion and hydrophobic connections. Hydrophobic interactions arise from stacking between the DNA nucleobases and the hydrophobic regions of the MWCNT surface, while electrostatic repulsion is due to the clash between the carboxylic groups of MWCNTs and the negative phosphodiester backbone of DNA. These differing electrostatic/hydrophobic properties lead to variations in the adsorption affinity of the ROX-L probe and dsDNA to the MWCNT surface, potentially enhancing the sensitivity and selectivity of the detection test. If the hydrophobic interactions outweigh the electrostatic repulsion, the DNA will adsorb onto the MWCNT surface (Figure 1) (48, 49). The biosensors technique was used to verify MWCNT nanoparticles and the MWCNT-ssDNA conjugate through SEM electron microscopy and EDX analysis. These characterization methods visually and chemically confirmed the presence of MWCNT nanoparticles and the successful conjugation of ssDNA onto them (44).

We suggest that this biosensing platform offers several advantages. This method can be applied in complex systems and avoids interference between Raleigh light scattering signals and dye fluorescence signals, thereby enhancing detection sensitivity. Consequently, it could eliminate the need for multiple laser excitation sources. Given the planar shape of MWCNTs and the simplicity of operation, the proposed method can adsorb a diverse array of DNA probes (50). Rafiee-Pour et al. developed an electrochemical biosensor capable of detecting miRNA-21 without the need for labeling, specifically aimed at identifying breast cancer (51). Salahandish et al. created an electrochemical nano-nanosensor using an NFG/AgNPs/PANI electrode combination to detect miRNA-21 cancer markers, which proved to be highly sensitive and specific (52). For detecting miRNA-21 expression in cancer cells, Liu et al. demonstrated the use of a fluorescent biosensor equipped with a 2-aminopurine (2-AP) probe alongside signal amplification (53). This biosensor amplifies the fluorescent signal in the presence of the target miRNA. Thanks to our enzyme-free signal amplification method, the sensor becomes easier and more cost-effective to use, potentially reaching a detection limit of 3.5 pM. This technique successfully identified the overexpression of miRNA-21 in human breast cancer cells. The proposed sensor could serve as a rapid and precise platform for detecting target miRNA, holding significant potential for the convenient monitoring of various miRNA biomarkers for the early detection of different cancers (53). Previous studies have indicated that miRNAs are modulated during the progression of colorectal tumors through overexpression, downregulation, or deletion (54).

Wang et al. developed a fluorescent biosensor for the detection of miRNA in live cells facilitated by MnO_2_ nanosheets. This method employed fluorescence resonance energy transfer (FRET) to detect miRNA-21, using FAM as the fluorescent donor and TAMRA as the fluorescent acceptor. This approach successfully differentiated the expression levels of miRNA-21 in HeLa and HepG-2 cells, highlighting the method's significant potential for early detection of diseases associated with miRNAs (36). Ji et al. introduced a fluorometric technique for the quantification and detection of miRNA, utilizing NMM and DAPI as fluorescent dyes for signal reporting. This technique led to a marked decrease in NMM fluorescence emission and an increase in DAPI fluorescence emission, enabling sensitive detection of miRNA. Importantly, by quantifying cancer-associated miRNA-125b and miRNA-21, this method demonstrated the capability to detect miRNAs with low and sub-picomolar detection limits, thereby facilitating miRNA analysis in biological materials through cell lysis (37). 

Wang et al. utilized quantum dots (CDs) and FAM-labeled ssDNA to construct a T7 exonuclease-mediated fluorescence biosensor for miRNA-21 detection. In the absence of miRNA-21, CDs absorbed and quenched the fluorescence of FAM-labeled ssDNA, but fluorescence emission was restored in the presence of miRNA-21. The sensor achieved a detection limit of 1 pM for miRNA-21, exhibiting selectivity and repeatability, and demonstrated a strong linear relationship between the amount of FAM/FCDs and miRNA-21 concentration within the range of 0.05 - 10 nM. Additionally, this sensor effectively measured the expression level of miRNA-21 in clinical blood samples from both healthy individuals and patients with gastrointestinal cancer (38). 

Sun et al. designed a fluorescent biosensor using DSN nuclease for miRNA detection aimed at diagnosing and treating acute pancreatitis (41). Although the results indicate high sensitivity and specificity for detecting specific miR-21-5p sequences, further investigation is needed, including optimization of the procedure (e.g., immobilization methods and hybridization) and the careful design of relevant DNA probes. In this study, a fluorescence sensing platform was developed to detect microRNA in plasma, potentially aiding in the diagnosis of CRC. Table 1 presents a comparison of this study with others, showing that the obtained results are promising. The research also suggests the potential to develop the proposed sensing platform into a highly selective, linear, multiplexed, and cost-effective system for miR-21 detection.

## Data Availability

The dataset presented in the study is available on request from the corresponding author during submission or after publication.
